# Multiomic Fermentation Using Chemically Defined Synthetic Hydrolyzates Revealed Multiple Effects of Lignocellulose-Derived Inhibitors on Cell Physiology and Xylose Utilization in *Zymomonas mobilis*

**DOI:** 10.3389/fmicb.2019.02596

**Published:** 2019-11-07

**Authors:** Yaoping Zhang, Jessica M. Vera, Dan Xie, Jose Serate, Edward Pohlmann, Jason D. Russell, Alexander S. Hebert, Joshua J. Coon, Trey K. Sato, Robert Landick

**Affiliations:** DOE-Great Lakes Bioenergy Research Center, University of Wisconsin–Madison, Madison, WI, United States

**Keywords:** synthetic hydrolyzates, lignocellulose-derived inhibitors, xylose utilization, multiomic fermentation, ethanol production, RND family efflux pump systems

## Abstract

Utilization of both C5 and C6 sugars to produce biofuels and bioproducts is a key goal for the development of integrated lignocellulosic biorefineries. Previously we found that although engineered *Zymomonas mobilis* 2032 was able to ferment glucose to ethanol when fermenting highly concentrated hydrolyzates such as 9% glucan-loading AFEX-pretreated corn stover hydrolyzate (9% ACSH), xylose conversion after glucose depletion was greatly impaired. We hypothesized that impaired xylose conversion was caused by lignocellulose-derived inhibitors (LDIs) in hydrolyzates. To investigate the effects of LDIs on the cellular physiology of *Z. mobilis* during fermentation of hydrolyzates, including impacts on xylose utilization, we generated synthetic hydrolyzates (SynHs) that contained nutrients and LDIs at concentrations found in 9% ACSH. Comparative fermentations of *Z. mobilis* 2032 using SynH with or without LDIs were performed, and samples were collected for end product, transcriptomic, metabolomic, and proteomic analyses. Several LDI-specific effects were observed at various timepoints during fermentation including upregulation of sulfur assimilation and cysteine biosynthesis, upregulation of RND family efflux pump systems (ZMO0282-0285) and ZMO1429-1432, downregulation of a Type I secretion system (ZMO0252-0255), depletion of reduced glutathione, and intracellular accumulation of mannose-1P and mannose-6P. Furthermore, when grown in SynH containing LDIs, *Z. mobilis* 2032 only metabolized ∼50% of xylose, compared to ∼80% in SynH without LDIs, recapitulating the poor xylose utilization observed in 9% ACSH. Our metabolomic data suggest that the overall flux of xylose metabolism is reduced in the presence of LDIs. However, the expression of most genes involved in glucose and xylose assimilation was not affected by LDIs, nor did we observe blocks in glucose and xylose metabolic pathways. Accumulations of intracellular xylitol and xylonic acid was observed in both SynH with and without LDIs, which decreased overall xylose-to-ethanol conversion efficiency. Our results suggest that xylose metabolism in *Z. mobilis* 2032 may not be able to support the cellular demands of LDI mitigation and detoxification during fermentation of highly concentrated lignocellulosic hydrolyzates with elevated levels of LDIs. Together, our findings identify several cellular responses to LDIs and possible causes of impaired xylose conversion that will enable future strain engineering of *Z. mobilis.*

## Introduction

Biofuel production from plant biomass provides a renewable and sustainable path to energy security. Native ethanologens, such as *Saccharomyces cerevisiae* and *Zymomonas mobilis*, can produce bioethanol and be engineered to produce advanced biofuels and bioproducts when biomass hydrolyzates are used without supplementation as the source of nutrients, carbon (mainly in the form of glucose), and energy (Lau and Dale, 2009; Lau et al., 2010; Jin et al., 2013; Skerker et al., 2013; He et al., 2014; Parreiras et al., 2014; Uppugundla et al., 2014; Serate et al., 2015; Yang et al., 2016; Nandy and Srivastava, 2018). Lignocellulosic hydrolyzates are primarily comprised of C6 and C5 sugar monomers, with glucose and xylose as the most abundant monomers respectively (Keating et al., 2014; Serate et al., 2015). However, neither *S. cerevisiae* nor *Z. mobilis* can natively metabolize the xylose that is abundant in lignocellulosic hydrolyzates.

Two xylose-utilizing strains of *Z. mobilis*, named *Z. mobilis* 2032 and *Z. mobilis* 8b, have been engineered by integration of *E. coli talB-tktA* and *xylA-xylB* genes into the chromosome or plasmids of wild-type *Z. mobilis* ZM4 (Zhang et al., 1995, 2007; Yang et al., 2018). Although both *Z. mobilis* 2032 and 8b can readily ferment xylose in rich medium (Zhang et al., 1995, 2007; Franden et al., 2013), xylose utilization remains slow, inefficient, and often incomplete in lignocellulosic hydrolyzates (Serate et al., 2015; Yang et al., 2018; Zhang et al., 2018). Several studies demonstrate that the high osmolarity of hydrolyzates, presence of lignocellulose-derived inhibitors (LDIs), and end-product toxicity can negatively affect microbial conversion of sugars in lignocellulosic hydrolyzates, especially xylose (Schwalbach et al., 2012; Keating et al., 2014; Tang et al., 2015; Zhang et al., 2018). Recently, we investigated the impacts of biomass feedstock variability on microbial conversion using both *S. cerevisiae* and *Z. mobilis* as model ethanologens (Ong et al., 2016; Zhang et al., 2018). Although *Z. mobilis* 2032 exhibits greater tolerance to LDIs than *S. cerevisiae* Y128, xylose-utilizing strains of both organisms struggle to metabolize xylose when grown in hydrolyzates with elevated LDIs (Ong et al., 2016; Zhang et al., 2018). For example, both *S. cerevisiae* Y128 and *Z. mobilis* 2032 utilize xylose poorly when grown in AFEX-pretreated corn stover hydrolysate (ACSH) from the year 2014 compared to ACSH from 2012 (Zhang et al., 2018). We identified elevated levels of LDIs in 2014-ACSH compared to 2012-ACSH, including ferulic and *p-*coumaric acids and feruloyl and coumaroyl amides. Adding these LDIs into 2012-ACSH to match 2014-ACSH levels inhibits xylose utilization to an extent resembling its use in 2014-ACSH (Zhang et al., 2018). Additionally, *Z. mobilis* 2032 exhibits poor xylose utilization when grown in highly concentrated 9%-glucan loading ACSH (9% ACSH) compared to 6%-glucan loading ACSH (Yang et al., 2018).

To provide consistent, but manipulable conditions for study of cellular responses to LDIs during fermentation of hydrolyzates, we developed synthetic hydrolyzates (SynHs) (Schwalbach et al., 2012; Keating et al., 2014; Serate et al., 2015; Tang et al., 2015). Our initial studies examined engineered, ethanologenic *E. coli* grown in SynH containing glucose, xylose, salts, amino acids, nucleobases, vitamins, and micronutrients at levels found in 6% ACSH (Schwalbach et al., 2012). In this SynH, *E. coli* exhibits stress responses induced by high osmolarity, LDIs, and ethanol. Further analysis of 6% ACSH led to a revised SynH version (SynH2) that includes osmoprotectants, acids, acetamide, additional sugars, and 14 LDIs, all added at concentrations found in 6% ACSH. We also created SynH2^–^ which lacks all LDIs (Keating et al., 2014). We used both SynH2^–^ and SynH2 along with 6% ACSH for systems-level studies of anaerobic growth of ethanologenic *E. coli*. Our studies identified several key regulators of cellular responses to LDIs that reduce the rate of ethanol production, especially during xylose conversion to ethanol (Keating et al., 2014).

Transcriptome profiling has been used to study the effects of some LDIs on *Z. mobilis* physiology. The majority of these studies focused on the effects of a single, abundant inhibitor, such as ethanol, acetate, furfural, or 5-hydroxymethylfurfural on *Z. mobilis* grown in rich or minimal media. However, the inhibitor concentrations used in these studies were often much higher than the concentrations found in real biomass hydrolyzates. Furthermore, cells were not subjected to the additional stresses of biomass hydrolyzates such as high osmolality, which exhibits synergistic inhibition with LDIs (He et al., 2012a, b; Yang et al., 2014b). To characterize the physiological impacts of LDIs on *Z. mobilis* in an industrially relevant context, we have developed a new version of SynH modeled on highly concentrated 9% ACSH, with and without added LDIs. We named these hydrolyzates SynH3 (with LDIs) and SynH3^–^ (without LDIs). We compared the cellular responses of xylose-utilizing *Z. mobilis* 2032 grown in SynH3^–^ or SynH3 by collecting and analyzing multiple omics-type data (multiomics) including transcriptomics, proteomics, and metabolomics. Our study focused on understanding how *Z. mobilis* responds to LDIs in SynH3 compared to SynH3^–^. We sought to identify the potential causes of impaired xylose conversion in the presence of LDIs. Addressing these effects will provide crucial information for engineering *Z. mobilis* strains with improved productivities of biofuels and bioproducts from lignocellulosic hydrolyzates.

## Materials and Methods

### Strains and Growth Media

Engineered xylose-utilizing *Z. mobilis* 2032 (ATCC31821-5C P*gap_taltkt*/P*gap xylAB*, PTA-6977) was obtained from American Type Culture Collection (ATCC). The rich media with glucose (ZRMG, containing 10 g yeast extract/L, 2 g KH_2_PO_4_/L, 20 g glucose/L) or rich media with both glucose and xylose (RMGX, containing 10 g yeast extract/L, 2 g KH_2_PO_4_/L, 100 g glucose/L, 20 g xylose/L) were used for initial cultivation of 2032. To increase buffer capacity and maintain pH, extra phosphate (0.8 g KH_2_PO_4_/L and 2 g K_2_HPO_4_/L) was added into RMGX, which was named as RMGXP.

### Preparation of SynH Media

Two synthetic hydrolyzate media used in these studies (SynH3^–^ and SynH3) were based on a previously described synthetic hydrolyzate medium (SynHv2.1) that mimics 6% glucan-loading AFEX-corn stover hydrolyzate (6% ACSH) (Serate et al., 2015). To mimic 9% ACSH, SynH3^–^ (Table 1) (aka SynHv3.8) was made by increasing the media composition of SynHv2.1 by 1.5-fold with the following additional modifications. To prevent Fe loss due to filtration of FeCl_3_ precipitate, FeCl_3_ was replaced with Fe-citrate, 1.5 mM sodium citrate was added, and 90 g/L of cellobiose was added to match the osmolarity of 9% ACSH (∼1920 mmol/kg). *Z. mobilis* 2032 is unable to metabolize cellobiose meaning that the osmolarity of SynH3^–^ will remain high throughout our fermentation experiments. We hypothesize that the lower osmolarity of SynH3^–^, prior to the addition of 90 g cellobiose/L, may be due to 9% ACSH components that are missing in our SynH recipe, likely stemming from an inability to identify or measure these compounds. This was an important modification as our previous work with *E. coli* highlighted osmotic stress as a key aspect of the stress response of *E. coli* to SynH and ACSH (Schwalbach et al., 2012; Keating et al., 2014). SynH3 (aka SynHv3.8) was generated by adding 25 LDIs (Table 2) to SynH3^–^ at LDI concentrations largely based on the composition analysis of 9% ACSH. All chemicals used for SynH, except two LDIs, were purchased from Fisher Scientific (Hampton, NH, United States) and Sigma-Aldrich (St. Louis, MO, United States). Feruloyl amide and coumaroyl amide were synthesized by in-house as described previously (Keating et al., 2014).

**TABLE 1:** Composition of SynH3^–^.

**Media component**	**SynHv2.1**^1^	**SynH3**^–^
**Carbohydrates (mM)**		
d-Glucose	333	500
d-Xylose	200	300
l-Arabinose	20	30
d-Galactose	2.9	4.4
d-Mannose	1.2	1.8
d-Fructose	24	36
**Misc. compounds (mM)**		
l-Lactic acid	4	6
Sodium nitrate	1.1	1.65
Sodium formate	2.8	4.2
Sodium succinate	0.5	0.75
Sodium acetate	32	48
Sodium citrate	−	1.5
Acetamide	80	120
Glycerol	4.1	6.15
Betaine	0.7	1.05
Choline chloride	0.3	0.45
dl-Carnitine	0.3	0.45
**Salts (mM)**		
KH_2_PO_4_	5.84	8.76
K_2_HPO_4_	11.15	16.725
KCl	36.8	55.2
NaCl	1.3	2
(NH_4_)_2_SO_4_	30	45
MgCl_2_	12.5	18.75
CaCl_2_	5.5	8.25
**Amino acids (μM)**		
Alanine	1172	1758
Arginine	144	216
Asparagine	228	342
Aspartate	594	891
Cysteine	50	75
Glutamine	259	388.5
Glutamate	607	910.5
Glycine	378	567
Histidine	37	56.1
Isoleucine	262	393
Leucine	371	556.5
Lysine	175	262.5
Methionine	100	150
Phenylalanine	282	423
Proline	656	984
Serine	369	553.5
Threonine	310	465
Tryptophan	50	75
Tyrosine	424	636
Valine	202	303
**Nucleobases (μM)**		
Adenine	50	75
Cytosine	50	75
Uracil	50	75
Guanine	50	75
**Vitamin, trace elements, and other components (μM or others indicated)**		
CuCl_2_	1.9	2.85
CoCl_2_⋅6H_2_O	0.03	0.045
H_3_BO_4_	23.1	34.65
(NH_4_)_6_Mo_7_O_2_⋅4H_2_O	0.31	0.465
FeCl_3_	20	−
Fe-citrate	−	30
ZnCl_2_	20	30
MnCl_2_⋅4H_2_O	91	136.5
Pyridoxine	2.14	3.21
Nicotinic acid	26.78	40.17
Thiamine-HCl	0.4	0.6
Pantothenate	3	4.5
Biotin	0.1	0.15
Inositol	56	84
Polysorbate 80 (Tween 80)	1 ml/L	1.5 ml/L
Ergosterol	10 mg/L	15 mg/L
**Increase osmolality to match 9% ACSH (1920 mmol/kg)**		
d-Cellobiose	−	90 g/L (263 mM)

*^1^Composition of SynHv2.1 was from paper published previously (Serate et al., 2015).− Not added in SynHv2.1 or SynH3^–^.*

**TABLE 2:** Lignocellulose-derived Inhibitors (LDIs) in SynH3.

**LDIs (μM)**	**SynH2**^1^	**SynH3**
**Amides**		
Feruloyl amide	2750	4230
Coumaroyl amide	2750	7130
4-Hydroxybenzamide	−	85
Syringamide	−	140
**Acids**		
*p*-Coumaric acid	1050	1440
Ferulic acid	355	180
Benzoic acid	480	320
Syringic acid	80	65
Cinnamic acid	90	135
Vanillic acid	90	10
Caffeic acid	10	15
4-Hydroxybenzoic acid	−	145
Azelaic acid	−	30
**Aldehydes, ketones, and related compounds**		
Vanillin	132	20
Syringaldehyde	162	20
4-Hydroxybenzeldehyde	197	220
4-Hydroxyacetophenone	25	15
Acetosyringone	−	20
Acetovanillone	−	20
Furfural	−	45
Hydroxymethylfurfural (HMF)	550	−
**Pyrazines and imidazoles**		
2-Methylimidazole	−	63
4(5)-Methylimidazole	−	129
2, 4-Dimethylimidazole	−	21
2-Methylpyrazine	−	9
2, 6-Dimethylpyrazine	−	1.5

*^1^Composition of LDIs in SynH2 was from paper published previously (Keating et al., 2014).- Not added in SynH2 or SynH3.*

### Fermentative Growth Conditions

Multiomic fermentations were conducted in 3 L bioreactors (Applikon Biotechnology) containing 1.2 L of SynH3^–^ or SynH3 media. Starter culture were first grown in ZRMG media with 12.5 μg/L each of Tetracycline (Tc) and Chloramphenicol (Cm) under aerobic conditions at 30°C. After ∼7 h, the cultures were diluted into RMGXP medium containing 12.5 μg/ml each of Tc and Cm at an initial OD_600_ (optical density at 600 nm) of 0.2, and then grown in an anaerobic chamber on a stir plate at 30°C for about 14 h. Cells from this culture were then centrifuged at 18000 × *g* for 3 min, cell pellets were resuspended in appropriate SynH media and inoculated into the bioreactors to give an initial OD_600_ of 0.5. Fermentations were conducted at 30°C with continuous stirring (300 rpm), headspace sparging with 100% N_2_ (20 mL/min) and pH controlled at 5.8. Samples were periodically removed from the bioreactors for an OD_600_ measurement using a Beckman Coulter DU720 (Beckman Coulter, Inc., Brea, CA, United State) to monitor cell growth and for YSI 2700 Biochemistry Analyzer (YSI, Inc., Yellow Springs, OH, United States) to monitor the concentration of glucose and xylose.

### Multiomic Sampling and Cell Dry Weight Measurement

During the fermentation, samples were collected at four growth stages: mid-glucose (about 50% glucose utilized), late-glucose (about 5–10 g glucose/L remaining), early-xylose (about 1 h after glucose was completely depleted) and mid-xylose (about 24 h after glucose was completely depleted) for transcriptomic, metabolomic, and proteomic analysis.

For RNA isolation and transcriptomic analysis, 10 ml of culture was collected in 15 ml conical tubes containing 1.25 ml ice-cold unbuffered phenol and ethanol mixture (5:95, vol/vol) and processed as described previously (Schwalbach et al., 2012).

For metabolomic sampling, 3–5 ml of culture was taken out from the bioreactor using 5 ml syringes. After quick capped the syringe, samples were transfer into an anaerobic chamber for subsequent processing to avoid the exposure of oxygen and the disturbance of metabolites. Samples were then loaded on a pre-wet 0.45-micron Nylon membrane filter (Whatman, Cat# 7404-002, 25 mm) under vacuum filtration (Hoefer, Holliston, MA, United States Cat# FH225V) to quickly separate the cells from the liquid medium. The membrane with cells was submerged into 3 ml ice-cold Extracting Solution containing acetonitrile:methanol:water (50:25:25) mixture and quickly vortexed. Samples were then removed from the anaerobic chamber and centrifuged at 18000 × *g* for 3 min at 4°C. One ml of supernatant was transferred in 1.5 ml Eppendorf tube, and quickly frozen in dry ice/ethanol bath and stored at −80°C.

For proteomic sampling, 2 ml of culture was collected and transferred into chilled 2 ml Microcentrifuge tubes. Cells were pelleted by centrifugation at 18000 × *g* at 4°C for 3 min, supernatant was removed, and cell pellets were quickly frozen in dry ice/ethanol bath for 5 min, and then stored at −80°C.

For dry cell weight (DCW) measurement, 25 ml of cells were collected, and after an OD_600_ measurement, 10–20 ml cells (total OD_600_ is about 30–40) were then loaded on a pre-wet 0.22 μM S-PaK membrane filter (Millipore, Cat# GSWG047S6, 47 mm) on the Nalgene filtration assembly (Nalgene, now Thermo Fisher Scientific, for 47 mm filter). The filter and cells were then washed with 5 ml Nanopure water and cells were dried in a desiccant chamber for 3–5 days and then weighed. The S-PaK membrane filters before the filtration were also pre-dried in desiccant chamber and weighed. The DCW values in milligrams per mL per OD_600_ of culture was calculated by the subtraction of the filter weight after filtration to that before filtration, and then normalized by volume of cells collected and its OD_600_.

### RNA Isolation and RNA-Seq Gene Expression Analysis

RNA was isolated from cells that were captured in phenol and ethanol solution as described previously (Rhodius and Gross, 2011). RNA samples were DNase treated and submitted to the University of Wisconsin Biotechnology Center (UWBC) Gene Expression Center for rRNA subtraction by Illumina RiboZero Bacteria Kit and paired-end library generation using the Illumina TruSeq Stranded Total RNA Library kit. Libraries were sequenced at 2 bp × 125 bp on an Illumina HiSeq2500 using v4 chemistry at the UWBC DNA Sequencing Facility. Reads were filtered for low quality and adapter readthrough using trimmomatic version 0.30 (Bolger et al., 2014) using the following parameters: ILLUMINACLIP:TruSeq3-PE.fa:2:22:10 SLIDINGWINDOW:4:28 MINLEN:75. Reads were aligned to *Z. mobilis* 2032 GenBank chromosome and plasmid sequences CP023677, CP023678, CP023679, CP023680, and CP023681 (Yang et al., 2018) using RSEM version 1.2.3 (Li and Dewey, 2011) and Bowtie version 1.0.0 (Langmead et al., 2009) using the following parameters: –paired-end –calc-ci –estimate-rspd –forward-prob 0 –phred33-quals.

RSEM expected counts were used for downstream differential expression analysis. Pearson correlation between vectors of counts that belong to biological replicates was employed to detect outlier libraries. All the retained libraries had inter-replicate Pearson correlation of at least 0.95. Features representing rRNA and tRNA, and genes with count sums less 5000 across all remaining samples were removed from the matrix. Gene count normalization and differential expression testing was performed using DESeq2 version 1.14.1 (Love et al., 2014) run on R version 3.3.0. Biclustering analysis of gene counts was performed using backSPIN run on python 2.7 (Zeisel et al., 2015).

### Proteomic Sample Preparation, Quantitation, and Data Analysis

#### Lysis and Digestion

Cells pellets were resuspended in 1 mL 6M GnHCl. Cells were lysed and protein was precipitated by adding MeOH to 90% final concentration. Samples was centrifuged at 15,000 × *g* for 15 min. Supernatant was discarded and pellets were dried for ∼5 min. Protein pellets were resuspended in 200 μL 8M urea, 100 mM Tris pH 8.0, 10 mM TCEP, and 40 mM chloroacetamide followed by dilution to 2M urea with 50 mM tris pH 8. Trypsin was added at an approximate ratio of 50:1, and samples were incubated overnight at ambient temperature. The supernatant was desalted over a PS-DVB cartridge and dried down. Peptides were resuspended in 0.2% formic acid and final peptide yield was estimated with absorbance at 205 nm with a nanodrop system (extinction coefficient = 31).

#### LC-MS/MS

For each analysis 0.5 μg was loaded onto a 75 μm i.d. 30 cm long capillary with an imbedded electrospray emitter and packed with 1.7 μm C18 BEH stationary phase. Peptides were eluted with in increasing gradient of acetonitrile over 100 min. Eluting peptides were analyzed with an Orbitrap Fusion Lumos. Survey scans were performed at *R* = 240,000. Data dependent top speed (1 s cycle time) MS/MS sampling of peptide precursors was performed with dynamic exclusion set to 20 s on precursors with charge states 2 to 5. MS/MS sampling was performed by quadrupole isolation = 0.7 Da, fragmentation by HCD with NCE = 30, max inject time = 11 ms, AGC = 3 × 10^4^ and analyzed in the ion trap with the “turbo” scan speed.

#### Data Analysis

Raw files were analyzed with the Maxquant v1.5.8.3 software program. Spectra was searched using the Andromeda search algorithm against a *Z. mobilis* database built from GenBank accessions CP023677, CP023678, CP023679, CP023680, and CP023681 (Yang et al., 2018). The default settings were applied except, fragment ion tolerance was set to 0.4 Da, match between runs was enabled, and label free quantitation was toggled on with the LFQ and protein label minimum ratio counts set to 1. Methionine oxidation and cysteine carbamidomethylation were set as variable and fixed modifications, respectively. Data for proteins that were not quantified in all of at least one condition were excluded from further analysis. Using the data analysis package Perseus v1.6.1.1, raw LFQ values were log2 transformed, missing values were imputed, and fold changes were calculated. To evaluate statistical significance of ratios, two sample *t*-tests were performed with the permutation based on FDR multiple hypothesis correction.

### Measurement of Internal Metabolite Abundances

Several different methods were used for measuring intracellular metabolites: AEX-LC-MS and IP-LC-MS were used for most intracellular metabolites, HILIC-MS was used for extracellular amino acids and GC-MS was used for intracellular sugar compounds.

#### Intracellular Metabolites by Ion-Pairing Liquid Chromatography Mass Spectrometry (IP-LC-MS)

Samples were vortexed and centrifuged at 13,000 × *g* for 10 min at 4°C to pellet protein and particulates. 400 μL of sample were mixed with 20 μL of ^13^C-labeled *Z. mobilis* cell extract and added to a 1.5 mL Eppendorf tube and dried under vacuum. Samples were reconstituted in water, and centrifuged at 13,000 × *g* for 10 min at 4°C to pellet additional protein and supernatant was applied to an ACQUITY UPLC HSS T3 reversed-phase column (Waters Corporation) with guard cartridge with an Ultimate HPG-3400RS pump and WPS-3000RS autosampler (Thermo Fisher). The LC system was coupled to a TSQ Quantiva Triple Quadrupole mass spectrometer (Thermo Scientific) by a heated ESI source. The Ion Transfer Tub Temp and Vaporizer Temp was kept at 350°C. For targeted analysis, the MS was operated in single reaction monitoring (SRM) mode acquiring scheduled, targeted scans to quantify selected compound transitions. All transitions and collision energies were previously optimized by infusion of each compound. The retention time window ranged from 2.5 to 10.0 min and the Use Calibrated RF Lens option was selected. MS acquisition parameters were 0.7 FWHM resolution for Q1 and 1.2 FWHM for Q3, 0.5 s cycle time, 1.5 mTorr CID gas, and 3 s Chrom Filter. Raw files were processed using Xcalibur Quan Browser (v4.0.27.10, Thermo Scientific) with results exported and further processed using Microsoft Excel 2010. Quantification was achieved through use of an external calibration curve constructed from the analysis of aqueous metabolite standard mixtures spiked into 13C-labeled metabolite extract and final metabolite concentrations normalized by DCW of cell pellet collected for extraction.

LC-MS/MS analyses were performed using a randomized sample order. An Ultimate HPG-3400RS pump and WPS-3000RS autosampler (Thermo Fisher) were mated to an ACQUITY UPLC HSS T3 reversed-phase column (2.1 mm × 150 mm, 1.8 μm particle diameter, Waters Corporation) with guard cartridge. The autosampler was cooled to 4°C and the needle bottom offset was set to 5 mm to avoid the potential of introducing protein precipitated in the bottom of the autosampler vial. Mobile phase A was 18.2 MΩ-cm water containing 10 mM tributylamine and 15 mM acetic acid (pH ∼4.85). Mobile phase B was methanol and gradient elution was performed at 0.375 mL/min with the column heated to 45°C.

The gradient method for separation was as follows: 0.0 to 12.0 min (hold at 2% B), 12.0 to 13.0 min (2–25% B), 13.0 to 25.0 min (25–75% B) 25.0 to 25.5 (75–100% B), 25.5 to 26.5 min (hold at 100% B), 26.5 to 27.0 min (100–2% B), 27.0 to 29.5 min (hold at 2% B), 29.5 to 30.0 min (increase flow rate to 0.550 mL/min), 30.0 min to 35.0 min (hold at 2% B). The LC system was coupled to a TSQ Quantiva Triple Quadrupole mass spectrometer (Thermo Scientific) by a heated ESI source. The Ion Transfer Tub Temp was kept at 350°C as was the Vaporizer Temp. Sheath Gas was set to 22 units, Auxiliary Gas to 3 units, and Sweep Gas to 1 unit. Negative and positive spray voltages were static and set to 3800 and 4000 V, respectively. For targeted analysis, the MS was operated in SRM mode acquiring scheduled, targeted scans to quantify selected compound transitions. All transitions and collision energies were previously optimized by infusion of each compound. The retention time window ranged from 2.5 to 10.0 min and the Use Calibrated RF Lens option was selected. MS acquisition parameters were 0.7 FWHM resolution for Q1 and 1.2 FWHM for Q3, 0.5 s cycle time, 1.5 mTorr CID gas, and 3 s Chrom Filter.

Raw files were processed using Xcalibur Quan Browser (v4.0.27.10, Thermo Scientific) with results exported and further processed using Microsoft Excel 2010. Quantification was achieved through use of an external calibration curve constructed from the analysis of aqueous metabolite standard mixtures spiked into 13C-labeled metabolite extract.

#### Intracellular Metabolites by Anion-Exchange Liquid Chromatography Mass Spectrometry (AEX-LC-MS)

A tube of 13C-labeled metabolite extract from Pichia Pastoris (Cambridge Isotope Labs) was reconstituted in 2.5 mL of water. Ten microliters of 13C extract were mixed with 140 μL of sample in a 1.5 mL Eppendorf tube. Samples were vortexed and centrifuged at 13,000 × *g* for 10 min to pellet particulates. One hundred microliters of sample were added to a to a low volume autosampler vial with tapered insert.

Mixed calibration standards were created from 5 to 10 mM stock solutions of each metabolite and serially diluted to create a concentration range from 0.098 to 200 μM. Ten microliters of 13C extract was added to each 150 μL standard prior to bringing up to full volume. Samples and standards were stored at 4°C until queued for analysis.

LC-MS/MS analyses were performed using a randomized sample order. An Ultimate HPG-3400RS pump and WPS-3000RS autosampler (Thermo Fisher) were mated to an IonPac AS-11 HC strong anion-exchange analytical column (2 mm × 250 mm, 9 μm particle diameter, Thermo Scientific) with guard column. The autosampler was cooled to 4°C. The injection volume was varied from 2 to 10 μL. Mobile phase A was 1 mM NaOH and mobile phase B was 100 mM NaOH and were degassed and then blanketed with helium for the duration of the analysis. Gradient elution was performed at 0.350 mL/min. Column eluent was passed through a 2 mm AERS 500 suppressor operated via a reagent-free controller (RFC-10, Dionex) set to 100 mA. The suppressor was operated in external water regeneration mode with water delivered at 2.0 mL/min by an Agilent 1100 binary pump. The column was heated to 40°C. The multi-step gradient method for separation was as follows: 0.0 to 5.0 min (hold at 12.5% B), 5.0 to 10.0 min (12.5–20% B), 10.0 to 17.5 min (20–27.5% B), 17.5 to 22.0 min (27.5–42.5% B), 22.5 to 35.0 min (42.5–100% B), 35.0 to 43.0 min (100% B), 43.0 to 43.1 (100–12.5% B, increase flow rate to 0.400 mL/min), 43 to 50 min (hold at 12.5% B). The LC system was coupled to a TSQ Quantiva Triple Quadrupole mass spectrometer (Thermo Scientific) by a heated ESI source kept. The Ion Transfer Tub Temp was kept at 350°C as was the Vaporizer Temp. Sheath Gas was set to 16 units, Auxiliary Gas to 3 units, and Sweep Gas to 2 units. Negative spray voltage was static and set to 3800 V. For targeted analysis the MS was operated in SRM mode acquiring scheduled, targeted scans to quantify selected metabolite transitions. All transitions and collision energies were previously optimized by infusion of each metabolite. The retention time window ranged from 5 to 10 min and the Use Calibrated RF Lens option was selected. MS acquisition parameters were 0.7 FWHM resolution for Q1 and 1.2 FWHM for Q3, 1 s cycle time, 1.5 mTorr CID gas, and 3 s Chrom Filter.

Raw files were processed using Xcalibur Quan Browser (v4.0.27.10, Thermo Scientific) with results exported and further processed using Microsoft Excel 2010. Quantification was achieved through use of an external calibration curve constructed from the analysis of aqueous metabolite standard mixtures.

#### Intracellular Amino Acids by Hydrophilic Interaction Liquid Chromatography Mass Spectrometry (HILIC-MS)

Samples were vortexed and centrifuged at 13,000 × *g* for 10 min to pellet particulates. Eighty microliters of sample was mixed with 100 μL 100 mM ammonium formate with 1.5% formic acid, 60 μL water, 10 μL 13C/15N-labeled amino acids internal standard (125 μM, Cambridge Isotope Labs), and 750 μL ACN so that the final sample composition was 75% ACN in 10 mM ammonium formate with 0.15% formic acid with the internal standard at 12.5 μM. Samples were centrifuged at 13,000 × *g* for 10 min at 4°C to pellet particulates and then 150 μL of sample was transferred to a low volume autosampler vial with tapered insert. Mixed calibration standards were created from 20 to 40 mM stock solutions of each amino and serially diluted to create a concentration range from 0.39 to 200 μM in 75% ACN with 10 mM ammonium formate and 0.15% formic acid. Ten microliters of 13C/15N-labeled standard was added to each standard prior to bringing up to full volume and then standards were transferred to autosampler vials. Samples and standards were stored at 4°C until queued for analysis.

LC-MS/MS analyses were performed using a randomized sample order. An Ultimate HPG-3400RS pump and WPS-3000RS autosampler (Thermo Fisher) were mated to an ACQUITY UPLC BEH Amide HILIC column (2.1 mm × 150 mm, 1.7 μm particle diameter, Waters Corporation) with guard cartridge. The autosampler was cooled to 4°C. Mobile phase A was 90% ACN with 10 mM ammonium formate and 0.15% formic acid. Mobile phase aqueous 10 mM ammonium formate with 0.15% formic acid. Gradient elution was performed at 0.400 mL/min. The column was heated to 35°C. The gradient method for separation was as follows: 0.0 to 3.0 min (hold at 5% B), 3.0 to 7.0 min (5–25% B), 7.0 to 12.0 min (25–50% B) 12.0 to 13.0 (hold at 50% B), 13.0 to 13.5 min (50–5% B, flow rate increased to 0.6 mL/min) 13.5 to 18.5 min (hold at 5% B). The LC system was coupled to a TSQ Quantiva Triple Quadrupole mass spectrometer (Thermo Scientific) by a heated ESI source. The Ion Transfer Tub Temp was kept at 350°C as was the Vaporizer Temp. Sheath Gas was set to 22 units, Auxiliary Gas to 13 units, and Sweep Gas to 1 unit. Negative and positive spray voltages were static and both set to 3800 V. For targeted analysis, the MS was operated in SRM mode acquiring scheduled, targeted scans to quantify selected compound transitions. All transitions, collision energies, and RF Lens voltages were previously optimized by infusion of each compound. The retention time window ranged from 2.5 to 4.0 min and the Use Calibrated RF Lens option was not selected. MS acquisition parameters were 0.7 FWHM resolution for Q1 and 1.2 FWHM for Q3, 1 s cycle time, 1.5 mTorr CID gas, and 3 s Chrom Filter.

Raw files were processed using Xcalibur Quan Browser (v4.0.27.10, Thermo Scientific) with results exported and further processed using Microsoft Excel 2010. Quantification was achieved by first generating an external calibration curve constructed from the analysis of aqueous amino acid standard mixtures along with labeled amino acid internal standards. A response factor was calculated for each amino acid relative to the internal standard and was used for quantification based upon the ratio of unlabeled amino acid to labeled internal standard in each sample.

#### Intracellular Sugar Metabolites by GC-MS

Samples were prepared without separation or filtering. Samples were thawed and vortexed to ensure thorough mixing. Preparation was done in National C4000-LV2W autosampler vials. First, 10 μL of each sample was dried using vacuum centrifugation. Then, 25 μL of a 20 mg/mL solution of *O*-methoxyamine-HCl in pyridine was added and the samples were vortexed and incubated at 37°C for 90 min. Next, 65 μL *N*-Methyl-*N*-(trimethylsilyl) trifluoroacetamide (MSTFA) was added, samples were vortexed, and incubated at 60°C for 30 min. Sample analysis was performed using a Trace 1310 GC coupled with an ISQ LT single quadrupole mass spectrometer (Thermo Scientific). The separation column used was a 30 m TraceGOLD TG-5SILMS (Thermo Scientific). Samples were injected in random order within each triplicate set (i.e., 951 was randomized, then 952, then 953). The gradient used was as follows: 0 to 2.00 min (hold at 150°C), 2.00 to 16.82 min (ramp 150–190°C), 16.82 to 18.12 min (ramp 190–255°C), 18.12 to 26.94 min (ramp 255–270°C), 26.94 to 27.94 min (ramp 270–320°C), 27.94 to 30.00 min (hold at 320°C). One μL was injected with a split ration of 1:10 for a nominal flow rate of 1.2 mL/min. The scan range used was 30–650 amu.

Raw files were processed with Thermo Scientific TraceFinder 4.0 General Quan software. Results were further processed with Microsoft Excel 2016. The external calibration curve comprised sugars at concentrations ranging from 0.2 to 500 μM. Linear regression curves formed using the standards allowed the back calculation of sample concentration using intensities. Height was determined to be the best method to quantify using this assay because most sugar species are not baseline resolved which leads to difficulty performing accurate peak area integration. Chromatographic co-elution and isobaric fragment ions shared between xylulose and ribulose posed a significant challenge to quantification. However, we observed two peaks when analyzing ribulose alone, though not baseline separated, and only one peak when running xylulose alone. Using this, we estimate ribulose contributes to the signal of xylulose by approximately 10–20% of total xylulose signal.

### Database Submissions and Accession Numbers

RNA-Seq data are available through the National Biotechnology Center Gene Expression Omnibus under accession number GSE135718. The mass spectrometry proteomics data have been deposited to the ProteomeXchange Consortium via the PRIDE (Perez-Riverol et al., 2019) partner repository with the dataset identifier PXD015007.

## Results

### Synthetic Hydrolyzates Largely Recapitulated the Growth and Sugar Consumption of *Z. mobilis* 2032 in 9% ACSH

**FIGURE 1:**
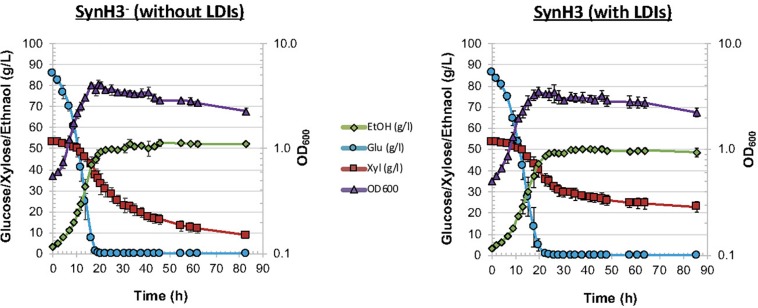
Fermentation of *Zymomonas mobilis* 2032 in SynH3^–^ and SynH3. Plots display cell growth by OD_600_ (purple triangles), glucose uptake (blue circles), and xylose uptake (red squares), and ethanol production (green diamonds) for comparative batch fermentations in SynH3^–^ and SynH3. Error bars denote standard deviation of mean values across three biological replicates.

As described in Section “Materials and Methods,” we developed SynH3^–^ to mimic of highly concentrated 9% ACSH. Twenty-five LDIs were added to SynH3^–^ to create SynH3 (Tables 1, 2). This created a system in which we could directly compare the impacts of LDIs on the physiological responses and fermentative capabilities of *Z. mobilis* 2032. To this end we performed batch fermentations of *Z. mobilis* 2032 in both SynH3^–^ (i.e., without LDIs) and SynH3 (i.e., with LDIs) in biological triplicates. Cell density (by light scattering), glucose, xylose, and ethanol production were monitored for 85 h (Figure 1). Because *Z. mobilis* 2032 is unable to co-metabolize both glucose and xylose, our fermentations resulted in a characteristic biphasic growth pattern in which glucose was metabolized first followed by xylose once glucose was nearly depleted. Extracellular ethanol reached a maximal concentration of approximately 50 g/L (5%) in both SynH3 and SynH3^–^ once glucose was depleted. During glucose fermentation *Z. mobilis* 2032 showed similar growth, glucose uptake, and ethanol production in both SynH3 and SynH3^–^ and was comparable to fermentation of *Z. mobilis* 2032 in 9% ACSH (Yang et al., 2018). One significant difference was that a growth lag was observed in 9% ACSH, whereas no lag was seen in either SynH media (Yang et al., 2018). In contrast, although more than 80% of xylose was consumed in SynH3^–^ after 85 h, only about 50% of xylose was utilized in SynH3. This result is consistent with previous observations in which xylose utilization was notably reduced when *Z. mobilis* 2032 was grown in 9% ACSH (Yang et al., 2018). These results indicated that growth in SynH3 was able to recapitulate the LDI-induced inhibition of xylose utilization that was observed in 9% ACSH.

These results indicated that LDIs do not appear to cause cell death, since glucose consumption and cell growth were not significantly different between SynH3^–^ and SynH3 fermentations. It is also possible that additive or synergistic effects of ethanol with LDIs on xylose utilization occur in our experiments. Previous studies investigating the effects of ethanol toxicity on *Z. mobilis* have observed negative impacts on cell growth and glucose consumption in *Z. mobilis* ZM4 in the presence of 5% ethanol in rich medium (He et al., 2012a). Our SynH3^–^ and SynH3 fermentations reached approximately 5% ethanol after glucose depletion; however, in the absence of LDIs, 5% ethanol does not inhibit xylose metabolism.

**FIGURE 2:**
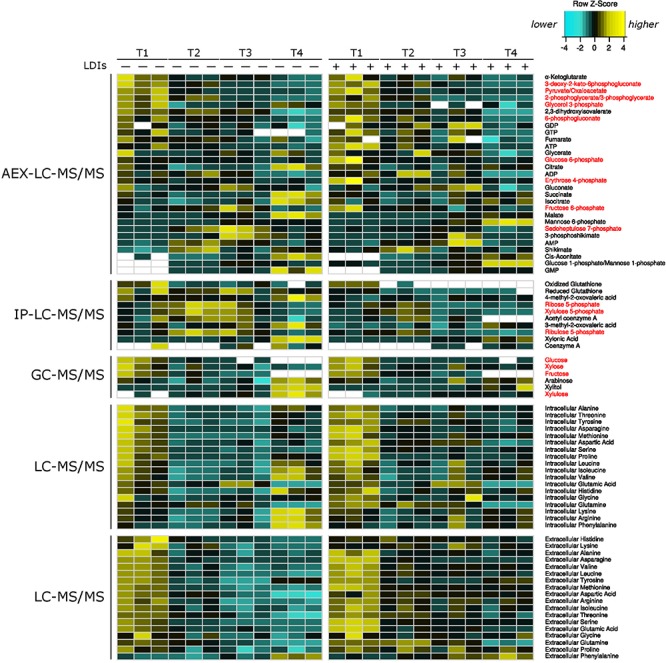
Heatmap of all metabolomics data. Heatmap of relative metabolite concentrations (rows) for each condition (columns) where metabolite concentration is scaled within each row. Yellow denotes higher metabolite levels and turquoise denotes lower metabolite levels, white denotes metabolite concentrations below the range of our standard curves. All concentrations were calculated as μmol/mg of dry cell weight for intracellular metabolites and as μM for extracellular amino acids. Metabolite names in red correspond to pentose phosphate and Entner–Doudoroff glycolysis metabolites. AEX-LC denotes anion exchange liquid chromatography and IP-LC denotes ion pair reversed-phase liquid chromatography.

To characterize the cellular responses of *Z. mobilis* 2032 to SynH3^–^ and the LDIs present in SynH3, we collected samples for metabolomic, proteomic, and transcriptomic analyses at four different timepoints during our batch fermentations: mid-glucose (T1, about 50% glucose utilized), late glucose (T2, about 5–10 g glucose/L remaining), early xylose (T3, ∼1 h after glucose was completely depleted), and late xylose (T4, ∼24 h after glucose was completely depleted). Using multiomics sampling, we were able to profile the relative expression levels of 1699 genes by RNA-Seq (Supplementary Table S1), 1587 proteins by LC-MS/MS proteomics (Supplementary Table S2), and using absolute levels, 63 intracellular and extracellular metabolites, including 18 intracellular and extracellular amino acids (Figure 2 and Supplementary Table S3).

### RNA-Seq Revealed a Complex and Dynamic Transcriptional Response to LDIs

**FIGURE 3:**
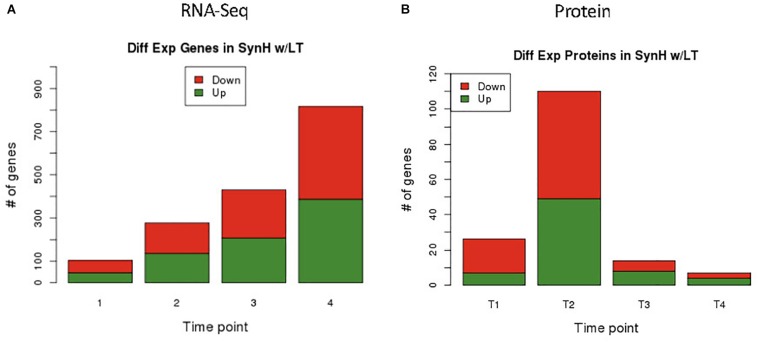
Differential expression results of genes by RNA-Seq **(A)** and proteins by LC-MS/MS **(B)**. From RNA-Seq data we identified 105, 287, 431, 818 differentially expressed genes in T1, T2, T3, and T4 respectively. Minimum FDR values for RNA-Seq statistical significance were set as follows: T1 FDR < 0.02, T2 FDR < 7.3E-3, T3 FDR < 4.7E-3, and T4 FDR < 2.45E-3. Different FDR values were selected to keep potential false positive hits to less than one for all comparisons. From proteomics data we identified 27, 111, 15, and 8 differentially expressed proteins in T1, T2, T3, and T4 respectively using a static FDR < 0.05.

**FIGURE 4:**
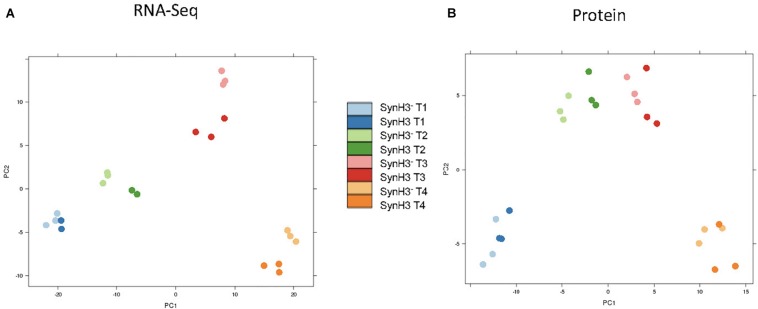
Principal coordinate analysis (PCA) plots of transcriptomic **(A)** and proteomic data **(B)**. Samples are color coded by timepoint and media. There were 2–3 biological replicates in each condition.

**FIGURE 5:**
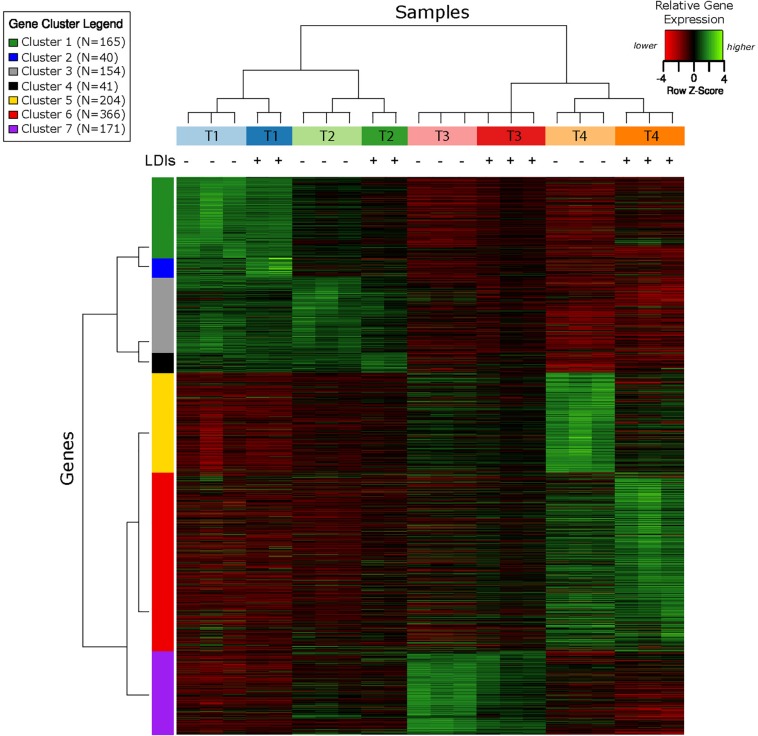
Bi-clustering analysis of gene expression reveals seven distinct clusters of gene expression patterns. Heatmap of relative gene expression (rows) for each condition (columns) where gene expression is scaled within each row. Samples are color coded by timepoint and media, with + or – additionally used to indicate the presence of LDIs in each sample. There were 2–3 biological replicates in each condition. The seven gene clusters are labeled with different colors.

RNA-Seq transcriptomics identified numerous differentially expressed genes between SynH3 and SynH3^–^ during the course of the fermentations. Overall, the number differentially expressed genes increased with subsequent timepoints and almost all genes in *Z. mobilis* 2032 were found to be differentially expressed at atleast one timepoint (Figure 3A). In contrast, our proteomics data identified far fewer proteins as differentially expressed, with T2 (about 5–10 g glucose/L remaining) having the highest number of differential protein levels (Figure 3B). Principal component analysis (PCA) of transcriptomic and proteomic data revealed that sample timepoint accounted for the greatest variance across samples, with biological replicates forming tight and distinct clusters in the transcriptomic data (Figure 4). Bi-clustering of transcriptomic data was consistent with PCA and produced distinct clustering by timepoint, with biological replicates clustering together into distinct clades at each time point (Figure 5). A total of seven gene clusters were identified, with each cluster corresponding to the timepoint and condition at which the highest expression of those genes was observed across all timepoints. Gene ontologies (GO) and KEGG pathways were used for gene-set-enrichment analysis (GSEA) of each cluster via hypergeometric tests and Holm multiple hypothesis testing correction (Supplementary Table S4). GSEA showed that transcription in both SynH3^–^ and SynH3 at T1 was enriched for growth genes such as ribosomal proteins and aminoacyl-tRNA biosynthesis, which is consistent with effects on glucose metabolism and logarithmic growth at this timepoint. At T1, SynH3 samples (gene cluster 2) also showed enrichment for sulfur, purine, seleno-compound metabolism, and aminoacyl-tRNA biosynthesis KEGG pathways. At T4, SynH3^–^ samples (gene cluster 7) were highly enriched for glycolysis–gluconeogenesis KEGG pathway and to lesser extent cell redox homeostasis and proteolysis GO terms. Our GSEA broadly highlights various impacts of LDIs at early glucose stages of fermentation and potential key differences in cellular activity between SynH3 and SynH3^–^ later when xylose was the primary carbon source.

### LDIs Induced Upregulation of Sulfur Assimilation Pathway Genes at Mid-Glucose (T1) Stage

At T1 approximately 100 genes were identified by RNA-Seq as differentially expressed in SynH3 relative to SynH3^–^ (at an FDR < 0.02; Supplementary Table S1). T1 differentially expressed genes are highly enriched for upregulation of KEGG sulfur metabolism pathway (KO00920). Most of the top 30 upregulated genes at T1 fall into one of four gene clusters: ZMO0003 to ZMO0009 (*cysCNDG1HIJ*), ZMO0282-0285 (efflux transporter), ZMO1260-1265 (*ssuACB* and others), and ZMO1459-1463 (*sseA* and others) (Supplementary Table S5).

ZMO0003 to ZMO0009 contains genes encoding cysteine biosynthetic proteins (sulfate assimilation). In *E. coli*, upregulation of *cys* genes is induced by sulfur limitation (van der Ploeg et al., 2001). Several other upregulated genes also indicate a disruption of sulfur homeostasis by LDIs in SynH3 including ZMO1261-1263 (*ssuACB*), ZMO1460 (sulfurtransferase), ZMO0055 (sulfite exporter) and ZMO0748 (cysteine synthase) (Supplementary Table S5). Upregulation of *cys* genes was also observed in *E. coli* grown in SynH containing LDIs (Keating et al., 2014). Miller et al. (2009) also found that the expression of genes and regulators associated with the biosynthesis of cysteine and methionine was increased by furfural treatment in *E. coli*. Most of the T1 upregulated genes involved in the biosynthesis of cysteine (sulfate assimilation) were only upregulated at this timepoint and returned to levels equivalent to non-LDI challenged cells at subsequent timepoints. These results suggest that disruption of sulfur homeostasis is an early and substantial effect of LDIs.

### RND Efflux Pump Systems Were Upregulated in the Presence of LDIs

**FIGURE 6:**
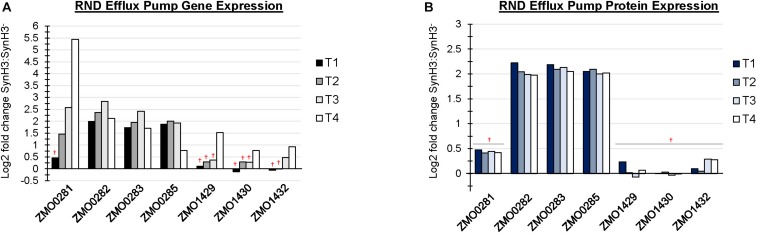
RND efflux pump systems were up regulated in the presence of LDIs. Log2 fold change comparing SynH3 to SynH3^–^ of Gene expression **(A)** and protein expression **(B)** of ZMO0281-0285 and ZMO1429-ZMO1432 RND efflux pump systems at all four timepoints (T1, T2, T3, and T4). ^†^Denotes not statically significant fold changes, all other fold changes are statistically significant.

There are at least four predicted efflux pump systems annotated in *Z. mobilis* 2032. The ZMO0282-0285 RND efflux transporter was upregulated at T1 and, together with ZMO0281, was also upregulated at T2, T3, and T4 (Figure 6A). ZMO0282, ZMO0283, and ZMO0285 protein levels were likewise elevated in SynH3 relative to SynH3^–^ at all four timepoints (Figure 6B). The RND efflux system at ZMO1429-1432 was also upregulated at T3 and T4 when xylose was the primary carbon source (Figure 6B). Both ZMO0282-0285 and ZMO1429-1432 encode RND family efflux transporter systems. In *E. coli* the AcrAB-TolC RND efflux pump is responsible for efflux of multiple antibiotics, dyes, bile salts, and detergents (Pradel and Pagès, 2002; Seeger et al., 2008; Li and Nikaido, 2009; Nikaido and Takatsuka, 2009). ZMO0281 encodes a TetR family transcriptional repressor of RND efflux transport system, which we hypothesize regulates ZMO0282-0285. As shown in Figure 6A, the expression of ZMO0281 was elevated from stage T2 to T4, and the high expression of ZMO0281 is correlated to the decreased expression of ZMO0282-0285 genes at T4. The transcriptional regulator of ZMO1429-1432 is unknown.

**FIGURE 7:**
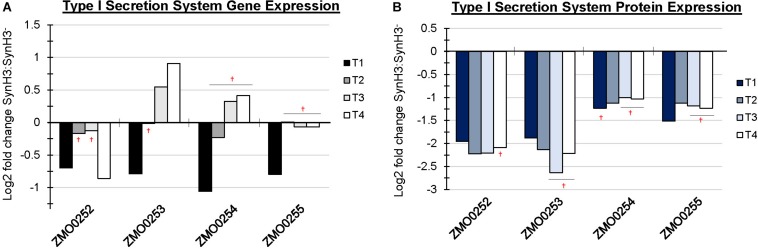
Operon containing Type I secretion system was downregulated at T1. Log2 fold change comparing SynH3 to SynH3^–^ of Gene expression **(A)** and protein expression **(B)** of ZMO0252-0255 (Type I secretion system) gene cluster at four different fermentation stages (T1, T2, T3, and T4). ^†^ Denotes not statically significant fold changes, all other fold changes are statistically significant.

In addition to upregulation of cellular efflux, LDIs also appear to impact expression of transporter and secretion systems in *Z. mobilis*. RNA-Seq identified the putative multidrug and toxic compound extrusion (MATE) efflux family protein (ZMO0214) as highly upregulated at T4 (Figure 6A). Additionally, five major facilitator superfamily (MFS) transporters (ZMO0099, ZMO0566, ZMO1111, ZMO1457, and ZMO2018) are either up or downregulated at T4. MFS transporters are single-polypeptide secondary carriers capable only of transporting small solutes in response to chemiosmotic ion gradients (Pao et al., 1998). Interestingly, transcriptomic data showed that all genes of the predicted Type I secretion system at ZMO0252-0255 were downregulated at T1 (Figure 7A). Proteomic data likewise showed the corresponding gene products to be highly downregulated at all four timepoints (Figure 7B). ZMO0253, ZMO0254, and ZMO0255 are proposed to encode a HylD family Type I secretion system and ZMO0252 encodes a major intrinsic protein.

### Mannose-1-Phosphate and Mannose-6-Phosphate Accumulated Intracellularly at T3 and T4

In our metabolomics data we observed an accumulation of intracellular mannose-6-phoshpate and mannose-1-phosphate over the course of our fermentations in both SynH3 and SynH3^–^. We note that our LC-MS assay is unable to distinguish between mannose-1P and glucose-1P, but given the concomitant accumulation of mannose-P, we reason that the change occurred primarily in mannose-1P, not glucose-1P. By T4, mannose-6P and mannose-1P reached 10.1 and 0.8 μmol per mg dry cell weight, respectively, in SynH3, which was > 4-fold higher than in SynH3^–^ at T4 (Figure 2, Supplementary Figure S1 and Supplementary Table S3). As there is no mannose-6P isomerase predicted in the *Z. mobilis* 2032 genome, mannose sugar phosphate accumulation likely occurred via uptake of D-mannose from the media since mannose is present in both SynH3 and SynH3^–^ at 1.8 mM. Phosphorylation of D-mannose may be catalyzed by two predicted *nagE* genes, ZMO0037 and ZMO1336 and further to D-mannose-1P by phosphomannomutase (ZMO0339). Interestingly, we observed a 2.7-fold upregulation of mannose-1-phosphate guanylyltransferase (ZMO1233) in SynH3 at T4 which may be contributing to higher accumulation of mannose-1P and mannose-6P relative to SynH3^–^.

### Reduced Glutathione Pool Is Decreased in the Presence of LDIs

**FIGURE 8:**
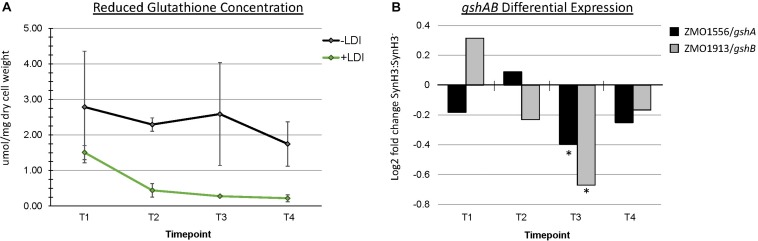
Reduced glutathione is markedly decreased in presence of LDIs. **(A)** Concentration of reduced glutathione, measured in μmol/mg of dry cell weight, from metabolomics across all four timepoints in SynH3^–^ (gray points and black line) and SynH3 (green points and line). Error bars represent standard error of the sample population with an n of 3. **(B)** Log2 fold change of glutathione biosynthetic genes *gshA* (black bars) and *gshB* (gray bars). ^∗^ Denotes statistical significance with an FDR < 0.0047.

The role of glutathione in detoxification of drugs, xenobiotics, and oxidants is well-documented (Forman et al., 2009). Metabolomics data revealed a striking depletion of reduced glutathione (GSH) specifically in SynH3 between T1 and later timepoints. In SynH3^–^ reduced glutathione was only depleted approximately 1.6-fold from T1 to T4, whereas in SynH3 reduced glutathione was depleted almost 7-fold (Figure 8A and Supplementary Table S3). At all four timepoints reduced glutathione was lower in SynH3 compared to SynH3^–^. Oxidized glutathione levels may have also been impacted by LDIs, however we could not accurately measure oxidized glutathione in SynH3 as levels were below the range of our standard curve. Glutathione is synthesized from glutamate, cysteine, and glycine in two steps catalyzed by GshA (ZMO1556) and GshB (ZMO1913). Both *gshA* and *gshB* expression were downregulated at T3 in SynH3 moderately, but statistically significantly (Figure 8B and Supplementary Table S1). Intracellular levels of glutamic acid were reduced > 4-fold at T4 relative to T1 in both SynH3^–^ and SynH3 while intracellular glycine was relatively unchanged during the course of our fermentations with a moderate increase in SynH3 at T3. We were unable to measure cysteine with our metabolomics assays.

### Several Stress Response Genes Are Upregulated at T3 and T4 in SynH3

Many genes were differentially expressed between SynH3 and SynH3^–^ at T3 and T4 (431 and 817, respectively; Figure 3A and Supplementary Table S1). The sheer number of differentially expressed genes, many of which are annotated as hypothetical proteins, makes it difficult to fully delineate the impact of LDIs during these xylose-metabolizing timepoints. However, several stress response genes were upregulated including *groES* (ZMO1928), *groEL* (ZMO1929), ZMO0199 (SOS-response transcriptional repressor, LexA), organic solvent tolerance protein (ZMO1311), and ZMO1996 (transcription termination factor Rho, which is known to respond to oxidative stress) (Supplementary Table S1). Downregulated genes at T4 included the ZMO0263-0270 gene cluster of hypothetical proteins and 44 plasmid-encoded genes (∼30% of total plasmid-encoded genes), many of which are predicted to encode transcriptional regulators.

### The Expression of Most Genes Involved in the Conversion of Glucose and Xylose Into Ethanol Was Not Affected by LDIs

**FIGURE 9:**
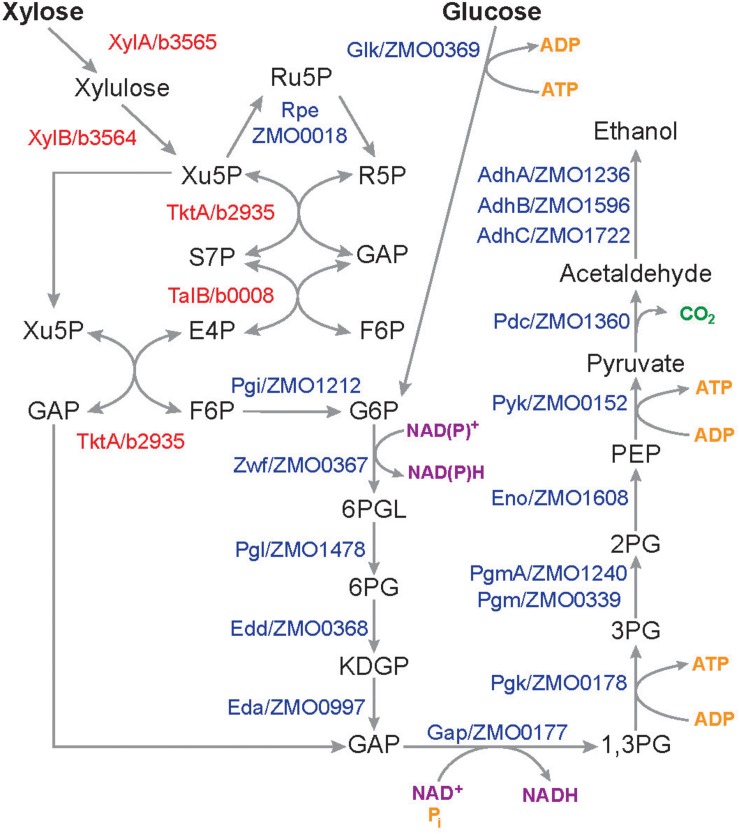
Pathways and genes involved in the conversion of glucose and xylose into ethanol in *Z. mobilis* 2032. Schematic showing pentose phosphate, Entner–Doudoroff glycolysis, and ethanol fermentation pathways. Protein names in red are *E. coli* genes that are heterologously expressed in *Z. mobilis* 2032. Xu5P, xylulose-5-phosphate; Ru5P, ribulose-5-phosphate; R5P, ribose-5-phosphate; GAP, glyceraldehyde-3-Phosphate; S7P, sedoheptulose-7-phosphate; E4P, erythrose-4-phosphate; F6P, fructose-6-phosphate; G6P, glucose-6-phosphate; 6PGL, 6-phosphoglucono-D-lactone, 6PG, 6-phosphogluconate; KDPG, 2-keto-3-deoxy-6-phosphogluconate; 1,3PG, 1,3-biphosphoglycerate; 3PG, 3-phosphoglycerate; 2PG, 2-phosphoglycerate; PEP, phosphoenolpyruvate.

The LDIs present in SynH3 have a consistent and profound negative effect on xylose metabolism by *Z. mobilis* 2032. We used our multiomics data to investigate how LDIs impact xylose metabolism in cells grown in SynH3. We compared the expression of all genes involved in the conversion of glucose and xylose to ethanol (Figure 9), including xylose-specific genes that are heterologously expressed in *Z. mobilis* 2032 (Zhang et al., 2007; Yang et al., 2018). Expression of glucose facilitated diffusion protein *glf*, which is involved in the uptake of both glucose and xylose, was also compared. With the exception of alcohol dehydrogenase *adhA*, there was no statistically significant difference in the expression of these genes or proteins in the presence of LDI at any timepoint (Supplementary Table S6). The expression of *adhA* was slightly down-regulated at T3 and T4. However, expression of two other alcohol dehydrogenases in *Z. mobilis* 2032, *adhB* and *adhC*, did not change, which could have compensated for reduced *adhA* expression. Thus, it is unlikely that slight down-regulation of *adhA* would cause the poor xylose utilization. These results indicated that the poor xylose utilization in SynH3 was not due to changes in the expression of sugar assimilation pathway genes.

### LDIs Did Not Create Bottlenecks in the Glucose and Xylose Assimilation Pathways

Although the expression of glucose and xylose metabolism genes was not affected by LDIs, the enzymes in these pathways may themselves be inhibited. We used our metabolomics to look for the accumulation of intermediate metabolites, which could indicate an enzymatic block in metabolism pathways. All metabolites in glucose and xylose assimilation pathways, except 6-phosphogluconate, 1,3-phosphoglycerate, phosphoenolpyruvate, acetaldehyde, and ethanol, were quantitated (Figure 9, Supplementary Figures S1, S2, and Supplementary Table S3). Many metabolites, including of 2-keto-3-deoxy-6-phosphogluconate, 2-phosphoglycerate, 3-phosphoglycerate, 6-phosphogluconate, fructose-6phophase, fructose, pyruvate, ATP, GTP, and glucose decreased from T1 to T4 in both SynH3 and SynH3^–^. However, there were no significant differences in the levels of these metabolites in SynH3 compared to SynH3^–^. Lower levels of these metabolites during xylose metabolism (T3 and T4) can be attributed to glucose depletion, slow xylose uptake, and reduced carbon flux. Levels of pentose phosphate pathway metabolites xylulose-5-phosphate, ribulose-5-phosphate, ribose-5-phosphate, sedoheptulose-7-phophate, and shikimate were elevated at T2, T3, or both, indicating flux from xylose metabolism, but ultimately these metabolites decreased by T4. Importantly, the levels of these metabolites were similar or slightly reduced in SynH3 compared to SynH3^–^. The levels of xylulose, xylonic acid, *cis-*aconitate, xylitol, and GMP increased in both SynH3^–^ and SynH3 from T1 to T4, but their levels were similar or higher in SynH3^–^ than SynH3 at T4 (Supplementary Figure S1). Greater consumption of xylose in SynH3^–^ likely contributes to the higher levels of these metabolites compared to SynH3. The production of xylonic acid and xylitol was an important contributor to reduced efficiency of xylose conversion to ethanol in the presence of LDIs.

## Discussion

Using a synthetic mimic of 9% glucan corn stover hydrolyzates, we directly probed the impacts of microbial inhibitors present in lignocellulosic hydrolyzates on *Z. mobilis* 2032 during batch fermentations. By collecting multiomic samples to quantitate transcript, protein, and metabolite levels, we found that the cellular response of *Z. mobilis* 2032 to LDIs is complex, and varied over the course of our fermentations. We observed multiple relevant changes including, but not limited to, upregulation of sulfur assimilation and metabolism, upregulation of RND efflux transporters and downregulation of a Type I secretion system, accumulation of mannose sugar phosphates, depletion of glutathione, and upregulation of general stress response genes in response to LDIs. Although we observed fewer differentially expressed proteins by proteomics analysis compared to transcriptomic changes, many of the differentially expressed genes and proteins have also been identified in previous studies of LDI stress responses (He et al., 2012a, b; Yang et al., 2014a; Yi et al., 2015). Transposon-enabled chemical genomics has also been used characterize stress responses of *Z. mobilis* to biomass hydrolyzates and LDIs (Skerker et al., 2013; Zhang et al., 2018). Many of the genes identified by our study were also identified as hydrolyzate tolerance genes study (Skerker et al., 2013), further supporting a role for these genes in mitigating LDI toxicity.

The upregulation of the ZMO0282-0285 and ZMO1429-1432 RND family efflux transporter systems in SynH3 could be an effective method of detoxification be removing LDIs from the cytosol. Interestingly, Skerker et al. (2013) only identified the ZM01429-1432 RND efflux system as important for hydrolyzate tolerance. We only observed upregulation of ZMO1429-1432 by RNA-Seq during xylose-metabolizing stages (i.e., T3 and T4). Neither of these efflux systems were differentially expressed under ethanol or acetate stress (He et al., 2012a; Yang et al., 2014a). In contrast, furfural stress induced downregulation of ZMO0285, ZMO1429, ZMO1431, and ZMO1432 after 24 h (He et al., 2012b). However, both ZMO0282 and ZMO0283 were found to be upregulated in response to phenolic aldehydes, including 4-hydroxybenzaldehyde, syringaldehyde, and vanillin (Yi et al., 2015). Our SynH3 recipe included 4-hydroxybenzaldehyde, syringaldehyde, vanillin, and furfural. These results underscore the importance of efflux pumps in helping *Z. mobilis* alleviate LDI toxicity. Recent work in *E. coli* found that upregulation of the *cusCFBA* efflux system was able to increase osmotolerance (Xiao et al., 2017), suggesting that upregulation of efflux systems in *Z. mobilis* may similarly contribute to osmotolerance in addition to LDI detoxification.

A decrease in protein secretion also appears to be an important cellular response to LDIs as was indicated by downregulation of the Type I secretion system at ZMO0253-0255 at T1. Changes in the expression of this operon in response to ethanol, acetate, furfural, or other LDI stress are not reported (He et al., 2012a, b; Yang et al., 2014a; Yi et al., 2015). Type I secretion systems are involved in the secretion of proteins from the cytoplasm to the extracellular medium in a single step for diverse purposes, including nutrient acquisition or bacterial virulence. The secreted substrates of this system are unknown in *Z. mobilis* making it difficult to interpret this result, but one possibility is reduced availability of energy for protein secretion leading to downregulation of expression.

At T1 many sulfur assimilation and sulfur metabolism genes were upregulated including the *de novo* cysteine biosynthesis operon *cysCNDG1HIJ* (ZMO0003-0009). Several studies have reported upregulation of these and other sulfur metabolism genes in response to furfural, acetate, and phenolic aldehydes (He et al., 2012a; Yang et al., 2014a; Yi et al., 2015). Chemical genomics identified ZMO0003, ZMO007-0009, and the iron-sulfur cluster assembly accessory protein ZMO0429 as important for hydrolyzate tolerance (Skerker et al., 2013). Likewise, several studies in *E. coli* have demonstrated the importance of sulfur assimilation in response to a number of stresses including LDIs and radical oxidative species (Miller et al., 2009). Previously, we observed up-regulation of *cys* genes in *E. coli* grown in SynH containing LDIs and suggested the effect might be caused by the impact of LDIs on cellular energetics (Keating et al., 2014). In *Salmonella enterica* serovar Typhimurium, CysJIH are proposed to protect against reactive oxygen species (ROS) induced by antibiotics or other compounds (Álvarez et al., 2015). Sulfur assimilation stress, which is also critical for maintenance of iron-sulfur cluster formation, clearly is a proximate of LDIs in *Z. mobilis* as well as other bacteria.

Skerker et al. (2013) proposed that an increased demand for sulfite and cysteine may be connected to a decrease in glutathione availability when *Z. mobilis* is grown in lignocellulosic hydrolyzates. Consistent with this hypothesis, we observed an almost sevenfold decrease in reduced glutathione (GSH) between T1 and T4 for cells grown in SynH3 (Figure 8). Furthermore, GSH levels were lower in SynH3 compared to SynH3^–^ at all timepoints. An increased demand for GSH is consistent with the role of GSH in cellular responses to many toxic compounds and stresses (Ferguson and Booth, 1998; Carmel-Harel and Storz, 2000; Pompella et al., 2003). GSH is also important for maintaining redox balance in the cytoplasm and for post-translational modification of proteins. The decrease in intracellular GSH in cells grown in SynH3 relative to SynH3^–^ may arise from increased GSH export, increased GSH conjugation to proteins and other substrates, such as LDIs, downregulation of GSH biosynthetic genes, inhibition of biosynthetic proteins, or limited availability of precursor substrates. Although we were unable to measure intracellular cysteine in our samples, upregulation of *cysCNDG1HIJ* suggests a decrease cysteine pools, activated sulfur pools, or both. Thus cysteine limitation, along with a decrease in glutamic acid at T3 and T4, may be the key contributor to decreased GSH levels in SynH3. Interestingly GSH biosynthesis genes *gshA* and *gshB* were downregulated at T3 which likely contributed to decreased GSH levels. Although decreased *gshAB* expression may appear contradictory to GSH stress, *gshAB* may be downregulated in response to cysteine limitation, by decreased cellular activity, or both. Notably sulfur assimilation is one of the most energetically costly cellular processes, suggesting a simple possibility that energy stress resulting from cellular responses to LDIs may compromise sulfur assimilation and thereby impact sulfur-containing metabolites.

We also observed a marked intracellular accumulation of mannose-1P and mannose-6P at T3 and T4. Though mannose sugar phosphate accumulation occurred in both SynH3^–^ and SynH3, this accumulation was greater in SynH3 which may be attributed, in part, to upregulation of mannose-1-phosphate guanylyltransferase (ZMO1233) specifically in SynH3 at T4. Mannose uptake from the medium, and subsequent accumulation, likely occurs via facilitated diffusion by Glf (ZMO0366) which, in addition to glucose and xylose, has been shown to aid D-mannose uptake in *Z. mobilis* (Weisser et al., 1996). Accumulation of mannose sugar phosphates has not been previously reported for *Z. mobilis* grown in lignocellulosic hydrolyzates nor is mannose a metabolizable substrate of *Z. mobilis* 2032. In *E. coli*, increased levels of sugar phosphates are known to contribute to cellular toxicity via methylglyoxal production and other unknown mechanisms (Kadner et al., 1992). It is unknown if sugar phosphate accumulation causes toxicity in *Z. mobilis*, but if it is possible that mannose phosphate accumulation in SynH3 at T3 and T4 could exacerbate LDI toxicity, possibly to an extent that the cells cannot recover. Interestingly, Skerker et al. (2013) identified a putative methylglyoxal detoxification system (*gloAB)* at ZMO0759-0760 as important for hydrolyzate tolerance, but both these genes were downregulated at T4 in SynH3. Two additional *gloA* genes are predicted in *Z. mobilis* 2032. Although *Z. mobilis* appears to lack a methylglyoxal synthase gene, the presence of multiple *gloA* genes suggests otherwise and warrants further investigation into whether *Z. mobilis* can produce methylglyoxal, particularly in response to sugar phosphate accumulation. Introduction of phosphomannose-isomerase (*pmi*) into *Z. mobilis* 2032 could help to mitigate potential toxicity due to mannose sugar phosphate accumulation while also providing an additional carbon source for ethanol production (Weisser et al., 1996).

Xylose is the most abundant C5 sugar in lignocellulosic biomass hydrolyzates. Complete conversion of xylose is an important requirement for an industrial biorefinery. Here, we demonstrated that LDIs are a major contributor to poor xylose utilization in *Z. mobilis* 2032 through the use of synthetic hydrolyzates with and without LDIs added. Although we have identified several cellular responses of *Z. mobilis* 2032 to LDI stress, the mechanisms by which LDIs specifically inhibit xylose utilization in *Z. mobilis* remain elusive. In *E. coli*, Pisithkul et al. (2015) found that feruloyl amide and coumaroyl amide act as potent and fast-acting inhibitors of purine and pyrimidine biosynthetic pathways. The block in *de novo* nucleotide biosynthesis leads to growth inhibition and subsequent cessation of carbon metabolism. However, we did not observe LDI-specific impacts on the expression patterns of glucose or xylose metabolizing genes in *Z. mobilis* nor did we observe any metabolic blocks in these pathways.

Overall, xylose flux appeared to be lower in SynH3 compared to SynH3^–^. Less carbon flux would result in production of less ATP and NAD(P)H, which are needed to mitigate LDI toxicity. Decreased flux may be due to decreased xylose uptake, decreased metabolic processing of xylose, or both. Glucose and xylose uptake occur *via* facilitated diffusion by Glf (ZMO0366). *glf* expression decreases over the course of our batch fermentations, but *glf* is not differentially expressed between SynH3^–^ and SynH3 (Supplementary Tables S1, S6). Higher accumulation of mannose phosphates in SynH3 would require increased uptake of D-mannose *via glf*, which could limit *glf* availability for xylose and thus reduce xylose uptake. Alternatively, increased cellular efflux, *via* upregulation of ZMO0282-0285 and ZMO1429-1432, could contribute to xylose efflux, by competing with Glf for space in outer and inner membranes (IM), or both. Recent studies have found that increased expression of non-PTS carbohydrate transporters (e.g., xylose transporter) by growth on alternative carbon sources or overexpression of unrelated IM proteins competes with antibiotic efflux systems for inner and outer membrane space in gram-negative bacteria. In these studies, competition for IM space decreased efflux-mediated resistance to different antimicrobial compounds (Villagra et al., 2012; Hidalgo et al., 2018). Conversely, in *Z. mobilis*, efflux pump upregulation could affect xylose transport in an analogous manner since Glf may not be efficient at xylose transport.

Reduced xylose flux could also be driven by a decrease in cellular metabolism. The production of xylitol and xylonic acid observed at T3 and T4 in both SynH3^–^ and SynH3 reduced the efficiency of xylose conversion to ethanol and subsequent NAD^+^ renewal. Furthermore, xylitol production via xylose reductase (ZMO0976) consumes NAD(P)H (Agrawal and Chen, 2011). We recently reported that *Z. mobilis* 2032 contains a single adenosine insertion in ZMO096 that causes a frameshift and subsequent premature stop codon after 235 amino acids (Yang et al., 2018). The activity of truncated xylulose reductase is unknown but our results suggest the truncated ZMO0976 may still be active or that glucose-fructose oxidoreductase (ZMO0689) may be producing xylitol *in vivo* (Zhang et al., 2009). In *Gluconobacter*, glucose dehydrogenase oxidizes glucose to gluconic acid and xylose to xylonic acid. In some bacteria, two enzymes are involved: xylose dehydrogenase catalyzes the 1st step to convert xylose to D-xylono-γ-lactone and xylonolactonase converts it to xylonic acid. It is also suggested that ZMO1649 (Gluconolactonase) might convert xylose to xylonic acid (He et al., 2014), but an experimental test remains to be performed. A few glucose dehydrogenases were also found in *Z. mobilis*, but it is unknown if these enzymes could also catalyze the conversion of xylose to xylonic acid.

Given that *Z. mobilis* metabolism of glucose is largely unaffected by LDIs, metabolic changes accompanying a shift to xylose metabolism are likely impeding the ability of *Z. mobilis* to manage the toxic effects of LDIs. Sulfur assimilation is an energy intensive process, requiring 2 ATP, a reduced thioredoxin, and 3 NADPH per atom of sulfur that is activated and assimilated. Several studies in *E. coli* have proposed that cells lose reducing power due to consumption of NAD(P)H for detoxification of LDIs which in turn inhibits sulfur assimilation (Miller et al., 2009). When the high energy demands of detoxification and sulfur assimilation are considered, it seems likely that inefficient xylose metabolism is unable to support the cofactor and energy needs of *Z. mobilis* in the presence of high levels of LDIs. Methods for improving xylose uptake and subsequent conversion to ethanol in lignocellulosic hydrolyzates with high LDIs may help to overcome the metabolic burden of LDI toxicity. Our findings suggest several avenues for future efforts aimed at elucidating the mechanisms of LDI toxicity and at improving xylose uptake and conversion in *Z. mobilis*.

## Data Availability Statement

The datasets generated for this study can be found in the RNA-Seq data are available through the National Biotechnology Center Gene Expression Omnibus under accession number GSE135718. The mass spectrometry proteomics data have been deposited to the ProteomeXchange Consortium via the PRIDE (Perez-Riverol et al., 2019) partner repository with the dataset identifier PXD015007.

## Author Contributions

YZ, JV, TS, and RL designed the project, analyzed data, and wrote the manuscript with input from all authors. DX, JS, and EP generated synthetic hydrolyzate, synthesized LDIs, conducted fermentation experiments, and analyzed data. JR, AH, and JC performed metabolites and proteomic quantitation and data analysis. All authors read and approved the final manuscript.

## Conflict of Interest

The authors declare that the research was conducted in the absence of any commercial or financial relationships that could be construed as a potential conflict of interest.

## Supplementary Material

FIGURE S1 Metabolite changes over time course of batch fermentations. Heatmaps displaying the change in metabolite concentrations calculated as the log2 fold change (lfc) of metabolites at each timepoint relative to T1. T1-relative fold change values were calculated within a given medium such that only SynH3 values were compared to SynH3 T1 and only SynH3^–^ values were compared to SynH3^–^ T1. Yellow denotes positive fold change values and an increase in metabolite levels from T1, turquoise denotes negative fold change values and a decrease in metabolite levels from T1, dark gray squares denote that a fold change value could not be calculated due to missing values for that metabolite.

FIGURE S2 Metabolite changes in SynH3 relative to SynH3^–^ at each timepoint. Heatmaps displaying the log2 fold change (lfc) of metabolites in SynH3 relative to SynH3^–^ at each timepoint. Yellow denotes positive fold change values and an increase in metabolite levels in SynH3, turquoise denotes negative fold change values and a decrease in metabolite levels in SynH3, dark gray squares denote that a fold change value could not be calculated due to missing values for that metabolite.

TABLE S1 Compiled RNA-Seq differential expression results.xlsx.

TABLE S2 Compiled proteomics differential expression results.

TABLE S3 Compiled metabolomics results.

TABLE S4 Gene ontology and KEGG pathway gene set enrichment analysis of Bi-clustering gene clusters.

TABLE S5 Top list of SynH3 upregulated genes at T1 stage. Four different colors indicate four gene clusters.

TABLE S6 The expression of genes involved in the conversion of glucose and xylose into ethanol at four different stages.
