# Supplementary figures and images for: Multiomic Fermentation Using Chemically Defined Synthetic Hydrolyzates Revealed Multiple Effects of Lignocellulose-Derived Inhibitors on Cell Physiology and Xylose Utilization in Zymomonas mobilis
Source: Front Microbiol. 2019 Nov 7;10:2596. doi: 10.3389/fmicb.2019.02596 (PMC6853872; doi:10.3389/fmicb.2019.02596)

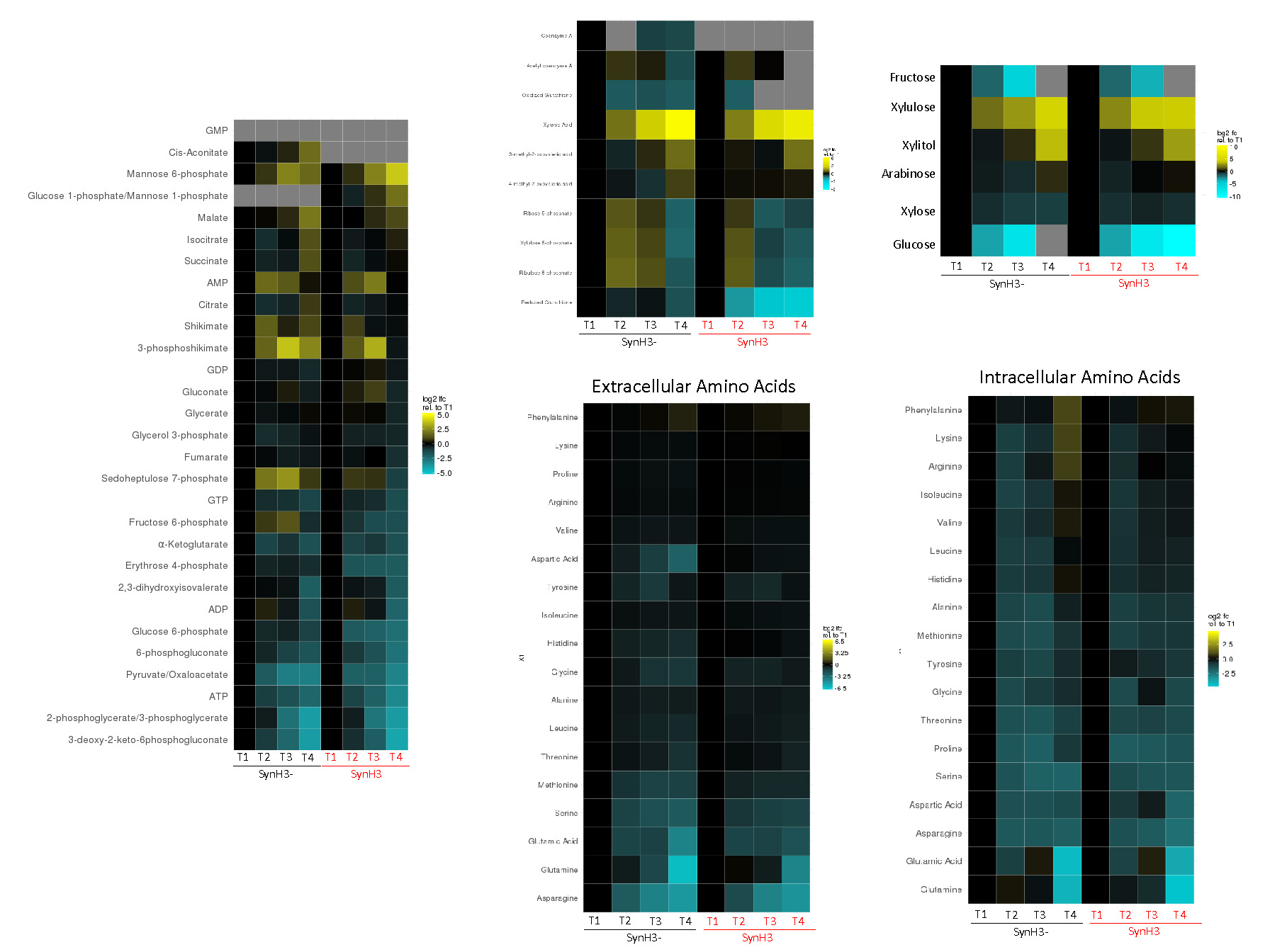

Supplement: FIGURE S1 — Metabolite changes over time course of batch fermentations. Heatmaps displaying the change in metabolite concentrations calculated as the log2 fold change (lfc) of metabolites at each timepoint relative to T1. T1-relative fold change values were calculated within a given medium such that only SynH3 values were compared to SynH3 T1 and only SynH3– values were compared to SynH3– T1. Yellow denotes positive fold change values and an increase in metabolite levels from T1, turquoise denotes negative fold change values and a decrease in metabolite levels from T1, dark gray squares denote that a fold change value could not be calculated due to missing values for that metabolite. [file Image_1.JPEG]

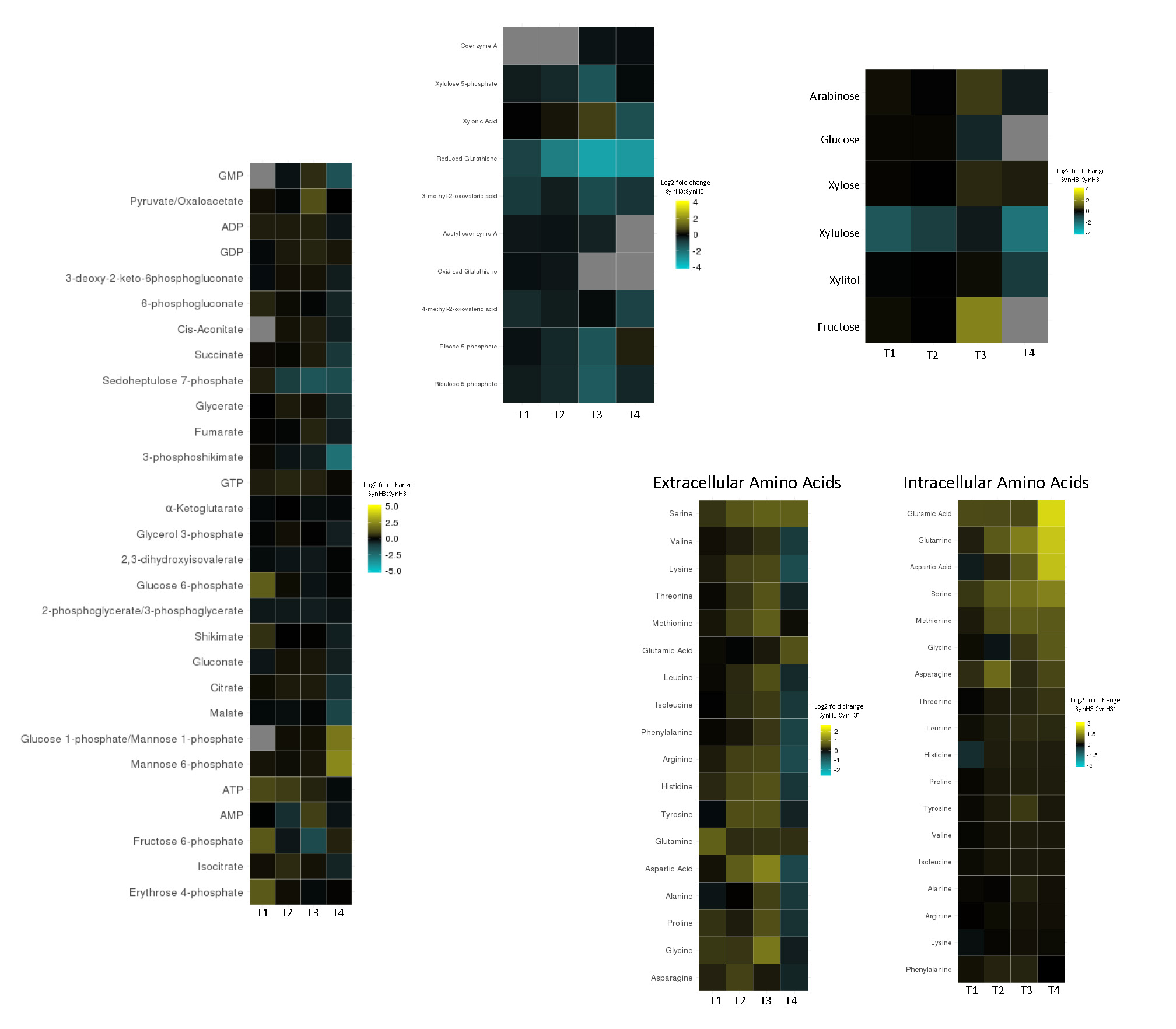

Supplement: FIGURE S2 — Metabolite changes in SynH3 relative to SynH3– at each timepoint. Heatmaps displaying the log2 fold change (lfc) of metabolites in SynH3 relative to SynH3– at each timepoint. Yellow denotes positive fold change values and an increase in metabolite levels in SynH3, turquoise denotes negative fold change values and a decrease in metabolite levels in SynH3, dark gray squares denote that a fold change value could not be calculated due to missing values for that metabolite. [file Image_2.JPEG]
